# Safety of natural anthraquinone emodin: an assessment in mice

**DOI:** 10.1186/s40360-021-00474-1

**Published:** 2021-01-28

**Authors:** Alexander T. Sougiannis, Reilly T. Enos, Brandon N. VanderVeen, Kandy T. Velazquez, Brittany Kelly, Sierra McDonald, William Cotham, Ioulia Chatzistamou, Mitzi Nagarkatti, Daping Fan, E. Angela Murphy

**Affiliations:** 1 Department of Pathology, Microbiology, & Immunology, School of Medicine, University of South Carolina, 6439 Garners Ferry Rd., Columbia, SC 29209 USA; 2 Department of Chemistry and Biochemistry, College of Arts and Sciences, University of South Carolina, Columbia, SC 29208 USA; 3 Department of Cell Biology and Anatomy, School of Medicine, University of South Carolina, Columbia, SC 29209 USA; 4 AcePre, LLC, Columbia, SC 29209 USA

**Keywords:** Toxicology, Pharmacology, Emodin, Sex differences, Alternative medicine

## Abstract

**Background:**

Emodin, a natural anthraquinone, has shown potential as an effective therapeutic agent in the treatment of many diseases including cancer. However, its clinical development is hindered by uncertainties surrounding its potential toxicity. The primary purpose of this study was to uncover any potential toxic properties of emodin in mice at doses that have been shown to have efficacy in our cancer studies. In addition, we sought to assess the time course of emodin clearance when administered both intraperitoneally (I.P.) and orally (P.O.) in order to begin to establish effective dosing intervals.

**Methods:**

We performed a subchronic (12 week) toxicity study using 3 different doses of emodin (~ 20 mg/kg, 40 mg/kg, and 80 mg/kg) infused into the AIN-76A diet of male and female C57BL/6 mice (*n* = 5/group/sex). Body weight and composition were assessed following the 12-week feeding regime. Tissues were harvested and assessed for gross pathological changes and blood was collected for a complete blood count and evaluation of alanine transaminase (ALT), aspartate transaminase (AST) and creatinine. For the pharmacokinetic study, emodin was delivered intraperitoneally I.P. or P.O. at 20 mg/kg or 40 mg/kg doses to male and female mice (*n* = 4/group/sex/time-point) and circulating levels of emodin were determined at 1, 4 and 12 h following administration via liquid chromatography with tandem mass spectrometry (LC-MS/MS) analysis.

**Results:**

We found that 12 weeks of low (20 mg/kg), medium (40 mg/kg), or high (80 mg/kg) emodin feeding did not cause pathophysiological perturbations in major organs. We also found that glucuronidated emodin peaks at 1 h for both I.P. and P.O. administered emodin and is eliminated by 12 h. Interestingly, female mice appear to metabolize emodin at a faster rate than male mice as evidenced by greater levels of glucuronidated emodin at the 1 h time-point (40 mg/kg for both I.P. and P.O. and 20 mg/kg I.P.) and the 4-h time-point (20 mg/kg I.P.).

**Conclusions:**

In summary, our studies establish that 1) emodin is safe for use in both male and female mice when given at 20, 40, and 80 mg/kg doses for 12 weeks and 2) sex differences should be considered when establishing dosing intervals for emodin treatment.

**Supplementary Information:**

The online version contains supplementary material available at 10.1186/s40360-021-00474-1.

## Background

Emodin (1,3,8-trihydroxy-6-methylanthraquinone) is a natural anthraquinone isolated from several Chinese herbs, including *Rheum palmatum, Polygonum cuspidatum,* and *Polygonum multiflorum*. Pre-clinical investigations have demonstrated emodin to contain a wide spectrum of pharmacological benefits, including; anti-viral [1], anti-bacterial [2], anti-allergic [3], anti-osteoporotic [4], anti-diabetic [5, 6], anti-inflammatory [7–10], neuroprotective [11], hepatoprotective [12], and anti-tumorigenic [13–18] properties (Supplementary Table 1). In fact, studies by our group have shown that emodin is effective at reducing mammary tumorigenesis given its actions on macrophages [14, 16]. Both the National Cancer Institute (NCI) and the National Center for Complementary and Integrative Health (NCCIH) recognize the importance of evidence-based complementary medicine modalities that may be integrated as part of standard cancer care for all patients across the cancer continuum. However, further development of emodin as an effective anti-cancer agent is hindered by uncertainties surrounding its potential toxicity.

Evaluation of the safety of dietary compounds is paramount to their clinical development. Several studies have reported side effects of emodin that may preclude its development beyond pre-clinical studies. For instance, it has been suggested that emodin may have mutagenic properties given the documented reports of genotoxicity and mutagenicity in certain strains of bacteria [19, 20]. There also are reports of hepatotoxic effects of emodin depending on the dose [21–25]. Further, at very high doses (1–3 g/kg/d for mice), emodin has been shown to have laxative effects leading to melanosis [20, 26]. While the number of negative reports is arguably balanced by the number of studies reporting no side-effects, uncertainties remain and have hampered enthusiasm for further development of this promising dietary agent.

Given the potential toxicity associated with emodin along with our efforts in the development of emodin as a complementary cancer therapy, we performed a sub-chronic toxicity study of emodin as a first step to clinical translation. We subjected mice to an emodin infused diet of three different concentrations (~ 20 mg/kg, 40 mg/kg and 80 mg/kg) – similar doses that we have used in our efficacy studies – for 12 weeks to monitor any potential toxic effects that emodin may have. In addition, we performed a pharmacokinetic study to evaluate the time course of emodin clearance from the circulation when given via two different routes of administration (intraperitoneally (I.P.) and orally (P.O.)). To ensure that we are not limiting translational relevance, both male and female mice were used in both the toxicity and pharmacokinetic studies.

## Methods

### Animals

Male and female C57BL/6 mice were purchased from Jackson Laboratories (Bar Harbor, ME) at 4 weeks of age and were cared for in the Department of Laboratory Animal Resources (DLAR) at the University of South Carolina’s School of Medicine. Mice (*n* = 4–5/group/sex/experiment) were randomized upon arrival to the DLAR to prevent litter biases. Mice were housed 4–5 per cage in a low-stress environment (22 °C, 50% humidity, low noise) and maintained on a 12:12-h light-dark cycle. All mice were handled only by the primary investigator (ATS). Mice were habituated to the AIN-76A diet (BioServ, Frenchtown, NJ, USA; catalog#:F1515) for 6 weeks prior to any experimental procedure (i.e. until 10 weeks of age). Experiments were initiated at 10 weeks of age and at average body weights of 27.57 ± 0.37 g for male and 19.73 ± 0.27 g for female mice. Food and water were provided ad libitum throughout the course of the study. All methods were performed in accordance with the American Association for Laboratory Animal Science and the Institutional Animal Care and Usage Committee at the University of South Carolina.

### Emodin

Emodin was purchased from Nanjing Zelang Medical Technology Co., Ltd., (Nanjing, China). Emodin was independently analyzed by the Mass Spectrometry Center at the University of South Carolina prior to the initiation of the experimental study. Liquid Chromatography -Ultraviolet-Mass Spectrometry (LC-UV-MS) and Nuclear Magnetic Resonance (NMR) was performed to confirm the purity and molecular structure of emodin.

### Emodin dosing and time course analysis for pharmacokinetic studies

At 10 weeks of age, emodin was delivered intraperitoneally (I.P.) or by oral gavage (P.O.) at 20 mg/kg or 40 mg/kg doses. For the I.P. study, emodin, dissolved in dimethyl sulfoxide (DMSO) (Spectrum Chemical, New Brunswick, NJ, USA; catalog#: BS580) was made in a large batch, aliquoted, and stored at -20 °C until used for injections. It was subsequently diluted in phosphate-buffered saline (PBS) (VWR, Suwanee, GA, USA; catalog# MRGF-6235-010Q) (1% DMSO) and administered to mice I.P. at doses of 20 mg/kg (0.5 mg/mL) or 40 mg/kg (1 mg/mL). To deliver emodin P.O., we utilized a 20G 30-mm flexible plastic tubal oral gavage needle (Instech, Plymouth Meeting, PA, USA; catalog#: FTP-20). To prevent aspiration of the emodin bolus, mice were briefly anesthetized with 2% isoflurane (provided by DLAR) prior to gavage and held upright until consciousness was regained. Emodin was prepared fresh on the day of administration. Briefly, emodin was mixed in pure propylene glycol (VWR, Suwanee, GA, USA; catalog# 97061) for 6–8 h at room temperature while protected from light. The emodin solution was delivered to mice at 20 mg/kg (6 mg/mL) and 40 mg/kg (12 mg/mL) doses. We used 1% DMSO in PBS (I.P.) or pure propylene glycol (P.O.) for vehicle controls.

Plasma emodin content was analyzed at three specific time points (t = 1 h, 4 h, and 12 h) after treating male and female wildtype (WT) *C57BL/6 J* mice with I.P. or P.O. administered emodin. Emodin was given at 20 mg/kg or 40 mg/kg; *n* = 4 mice were used per dosage, per timepoint, and per sex. Only a single dose of emodin was administered for the pharmacokinetic study. Vehicle treated mice were used to analyze empty plasma extracts.

### Solid phase extraction and liquid chromatography with tandem mass spectrometry (LC-MS/MS) analysis

Whole blood was collected in ethylenediaminetetraacetic (EDTA) (VWR, Suwanee, GA, USA; catalog#: 454428) coated tubes from the inferior vena cava and centrifuged at 4000 RPM for 10 min. Plasma was aliquoted and stored at -20 °C until solid phase extractions. For free emodin quantification, 50 μl of plasma was mixed with equal volume of 0.2 M sodium acetate buffer (Honeywell, Charlotte, NC, USA; catalog#: 71190) with 1% ascorbic acid (VWR, Suwanee, GA, USA; catalog#: BDH9242) (pH 5.0). For emodin glucuronide quantification, 50 μl of plasma was mixed with half the volume of 0.2 M sodium acetate buffer with 1% ascorbic acid and 1000 units of β-glucuronidase (Millipore Sigma, Burlington, MA, USA; catalog# G2174). D4-emodin (Santa Cruz Biotechnology, Dallas, TX, USA; catalog# 218302) (1 ng/μl) was used as an internal standard. Both tubes were then incubated at 37 °C for 2 h. After incubation, the mixture was extracted with 600 μl ethyl acetate (Fisher Scientific, Waltham, MA USA; catalog #: E195–4) three times. The ethyl acetate layer was evaporated under N_2_ gas to dryness and reconstituted in 5% ammonia water. Solid phase extraction was performed using a vacuum manifold (Millipore Sigma, Burlington, MA, USA; Supelco catalog#: SU57250-U) with Oasis MCX cartridges (Fisher Scientific, Waltham, MA USA**;** catalog #:186000254) and was eluted with 3 mL 5% formic acid-methanol (Fisher Scientific, Waltham, MA USA**;** catalog #s: A117–50 and A452–4). After elution, the eluent was evaporated under N_2_ gas to dryness and reconstituted in 400 μl 5% ammonia methanol (Fisher Scientific, Waltham, MA USA**;** catalog #s: A669–212 and A452–4).

Emodin samples were analyzed and quantified by LC-MS/MS using electrospray ionization in negative ion mode. Chromatorgraphic separation was performed on a Waters Acquity UPLC system using a binary solvent gradient. Solvent A was water containing 0.1% formic acid and solvent B was methanol. The LC column was a Waters XBridge C18 reversed phase column (2.1 mm X 100 mm containing 3.5um particles) running at a flow rate of 0.2 mL/min. The solvent gradient started at 50%B, ramped to 95%B over 10 min and was maintained at 95%B until 14 min. The gradient then returned to initial conditions. The mass spectrometer was a Waters Premier XE triple quadrupole instrument. Data was collected in multiple reaction monitoring (MRM) mode. Two precursor/product ion pairs were monitored; one pair for emodin (269 Da > 225 Da) and one pair for the internal standard deuterium labeled emodin (273 Da > 229 Da). According to the standard curve of emodin in 5% ammonia methanol, the concentration of emodin was calculated relative to the D4-emodin internal standard. Total emodin was measured from the tube containing the β-glucuronidase, free emodin was measured from the tube without β-glucuronidase, and glucuronidated emodin was calculated as the difference between total emodin and free emodin.

### Emodin diets for sub-chronic toxicity study

We analyzed the effect of emodin feeding over a 12-week period. Emodin was infused into the AIN-76A diet at three different concentrations; 170 mg/kg, 340 mg/kg, and 680 mg/kg. These concentrations were based on established average food intake of C57BL/6 mice and translate to a daily ingested dose per body weight of ~ 20 mg/kg, 40 mg/kg, and 80 mg/kg, respectively – doses that we have found to have efficacy in our cancer and cancer therapy studies. Briefly, mice were purchased at 4 weeks of age, kept on an AIN-76A diet until 10 weeks of age, and then separated into experimental groups and started on their respective diets as follows: at 10 weeks of age male and female mice were randomized into 4 groups consisting of *n* = 5 sex/diet including control (AIN), 20 mg/kg, 40 mg/kg, and 80 mg/kg. Mice were maintained on their treatment diets for 12 weeks and were given food and water ad libitum.

### Body weights and body composition

Body weight, food, and water consumption were monitored on a weekly basis for the duration of the study. Body composition was assessed after 12 weeks of emodin diet using dual-energy X-ray absorptiometry (DEXA) (Lunar PIXImus, Madison, WI, USA). Briefly, mice were placed under gas anesthesia (isoflurane, 2%) and were assessed for bone mineral density (BMD), lean mass, fat mass, and body fat percentage. Lean mass (%) was calculated as percent lean weight and bone mineral content (BMC) of total body weight.

### Tissue collection

After 12 weeks of dietary treatment, mice were euthanized by isoflurane overdose following a 4 h fast. Blood was collected from the inferior vena cava and placed in EDTA coated lavender top tubes for plasma collection and blood panel analysis. Spleen, liver, kidney, heart, colon, and ileum were harvested and fixed overnight in 10% neutral buffered formalin (VWR, Suwanee, GA, USA; catalog#:16004–128) and subsequently embedded in paraffin blocks. Colon and ileum were cleaned with PBS and swiss rolled prior to fixation. Liver, epididymal fat, mesenteric fat, and spleen weight were determined from freshly excised tissue prior to fixation. Colon, entire small intestine, and tibial length were also measured using calipers during tissue collection.

### Blood panel analysis

A complete blood panel analysis was performed using the VetScan HMT (Abaxis, Union City, CA, USA) for determination of white blood cells (WBC), lymphocytes (LYM), monocytes (MON), neutrophils (NEU), platelets (PLT), red blood cells (RBC), hematocrit (HCT), and hemoglobin (Hb). Neutrophil/lymphocyte ratio (NLR) was calculated from obtained values.

### Plasma markers of toxicity

Plasma was analyzed for common markers of major organ toxicity/physiological impairment including alanine transaminase (ALT) (Cayman Chemicals, Ann Arbor, MI, USA; catalog#: 700260), aspartate transaminase (AST) (Cayman Chemicals, Ann Arbor, MI, USA; catalog#: 701640), and creatinine (Cayman Chemicals, Ann Arbor, MI, USA; catalog#: 700460) and according to manufacturer’s instructions.

### Histopathology analysis

All tissues collected were stained with hematoxylin and eosin (H&E) (Fisher HealthCare, Irvine, CA, USA; catalog#: 245–651 and 245–827) as previously described by our group [27]. To analyze the presence of fibrosis, trichrome staining was performed in heart, liver, kidney, and spleen as previously described [28]. Goblet cells were identified in the colon by Alcian blue (Alfa Aesar, Haverhill, MA, USA: catalog#: J6012209) staining and counterstained by nuclear fast red as previously described [29]. Histological sections of the small intestine were examined for findings of inflammation (atrophy of the villi, infiltration of the lamina propria by inflammatory cells). Sections of the colon were evaluated for findings of colitis (destruction of the colonic mucosa, decreased number of goblet cells, and infiltration of the lamina propria by inflammatory cells) and/or dysplasia, graded as low or high dysplasia. Kidney, spleen and heart were evaluated with H&E staining for architectural and cellular abnormalities and for the presence of fibrosis (kidney and heart) with trichrome staining. For the histological examination of the liver specimens we used the scoring system designed by the Pathology Committee of nonalcoholic steatohepatitis (NASH) Clinical Research Network, which addresses the full spectrum of lesions of NAFLD [30]. alpha-Smooth Muscle Actin (α-SMA) (Cell Signaling, Danvers, MA, USA: #D4K97) was detected by immunohistochemical analysis in liver specimens using heat-based antigen retrieval rodent decloaker (Biocare, Pacheco, CA, USA: #RD913). Positivity was detected with goat anti-rabbit HRP-conjugated IgG secondary antibody (Abcam, Cambridge, UK: ab6721) and developed with DAB chromogen (Biocare, Pacheco, CA, USA: #BDB2004L). All histological analyses were performed blindly by a certified pathologist (I.C.).

### Statistical analyses

Analysis was performed using commercial software (SigmaStat V3.5, SPSS, Chicago, IL). All toxicity data were analyzed using a one-way analysis of variance (ANOVA). To examine sex differences in the pharmacokinetic data we performed a paired t-test at each time point. Any data that were not normally distributed or did not display equal variance were logarithmically transformed so that those criteria were met. Statistical significance was set with an alpha value of *p* < 0.05. Data are presented as mean ± standard error of mean (SEM).

## Results

### There is no difference in bioavailability of emodin when given I.P. vs P.O. but it is more bioavailable in female mice

**Fig. 1:**
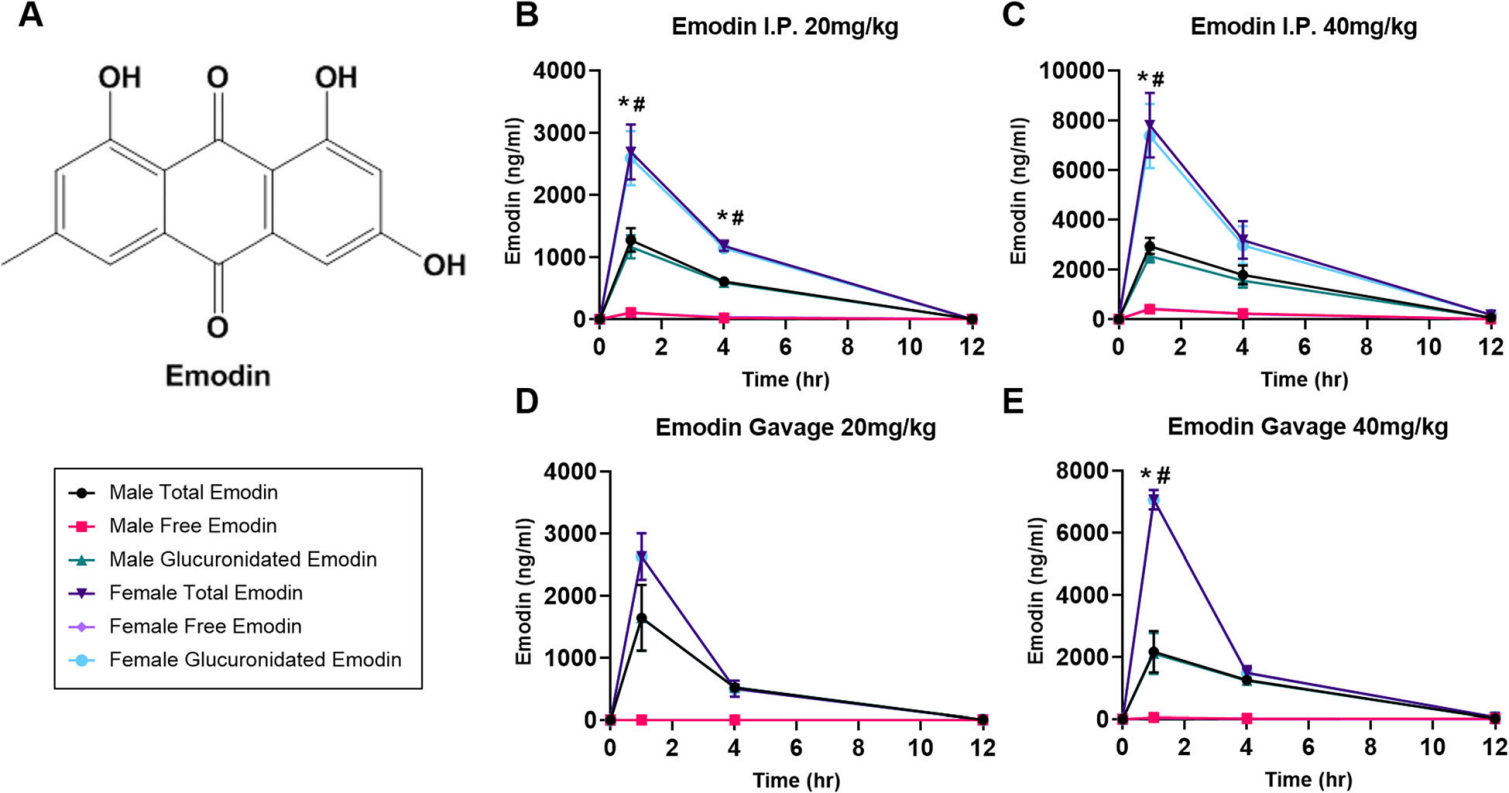
Emodin is rapidly glucuronidated when given I.P. and P.O. and is more bioavailable in females. **a**. Chemical structure of emodin (PubChem CID: 3220). **b-e.** Total, glucuronidated, and free emdoin bioavailability in male and female mice 1 h, 4 h, and 12 h after given I. P at **b**) 20 mg/kg and **c**) 40 mg/kg and P.O. at **d**) 20 mg/kg and **e**) 40 mg/kg. *n* = 3–4/dose/timepoint/sex. * indicates statistical significance (*p* < 0.05) between sexes of total emodin. # indicates statistical significance (*p* < 0.05) between sexes of glucuronidated emodin

We measured the concentrations of free emodin and glucuronidated emodin in plasma of mice given emodin via I.P. or P.O. at 20 or 40 mg/kg doses and at t = 1, 4, or 12 h (*n* = 4/timepoint/sex; Fig. 1b-e). The majority of emodin detected in each sample was glucuronidated while free emodin was scarcely found at all time points following both I.P. and P.O. routes of administration. The highest point of emodin detection was at the t = 1 h time point and was at approximately 50% concentration at t = 4 h and was completely eliminated at t = 12 h. Interestingly, we found that female mice had greater concentrations of glucuronidated emodin at t = 1 h in all doses and routes except for the 20 mg/kg dose given P.O. (Fig. 1b-e, *p* < 0.05) and at t = 4 h only in mice given the 20 mg/kg dose by I.P. (Fig. 1b, *p* < 0.05). Overall, there was no difference between I.P. and P.O. administration at any of the time points tested.

### Twelve-week emodin feeding does not present phenotypic toxicities in mice

**Fig. 2:**
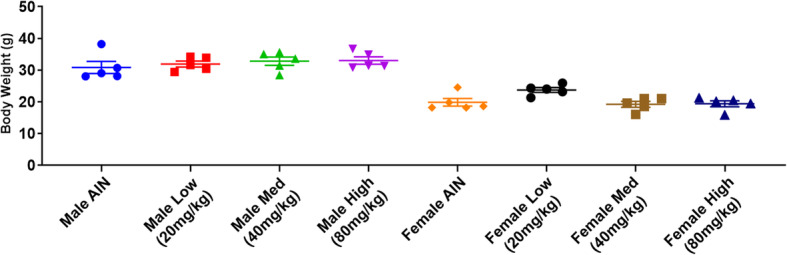
Twelve-week emodin feeding does not present phenotypic changes in male and female mice. **a**. Body weight at end of 12-week feeding for male and female mice. *n* = 5/group

We fed male (*n* = 5/group) and female (*n* = 5/group) mice a purified diet (AIN-76A) infused with three different concentrations of emodin. In statistical analysis, male and female mice were combined. After 12 weeks of feeding, emodin dose did not affect body weight compared to control mice (AIN-76A fed) (Fig. 2a). Similarly, body composition analysis did not show any differences in lean mass (%) or body fat (%) (Table 1) for any of the emodin doses versus the control group. Further, there was no difference in organ weight (liver, spleen, mesenteric fat, epididymal fat) or length (colon length, small intestine length, or tibial length) with emodin dose (Table 1).

**Table 1:** Male and female mice fed emodin for 12-weeks do not show differences in body composition and major organ weight. Lean mass and body fat percentage (%) were measured using DEXA scan. Liver, spleen, mesenteric fat, and epididymal fat were measured in milligrams (mg). Tibial length was measured in millimeters (mm). Colon length and small intestine (SI) length were measured in centimeters (cm). *n* = 5/group

Group	Lean Mass (%)	Body Fat (%)	Liver (mg)	Spleen (mg)	Mesenteric Fat (mg)	Epididymal Fat (mg)	Tibial Length (mm)	Colon Length (cm)	SI Length (cm)
**Control (AIN)**	72.5 ± 1.11	18.4 ± 1.65	1162.0 ± 90.66	110.1 ± 9.72	281.8 ± 41.55	464 ± 118.45	16.57 ± 0.15	8.0 ± 0.21	32.7 ± 0.58
**Low Emodin (20 mg/kg)**	72.8 ± 1.19	19.6 ± 1.21	1225.4 ± 67.54	110.1 ± 7.52	333.8 ± 39.18	6098.1 ± 86.46	16.65 ± 0.14	7.95 ± 0.17	32.35 ± 0.50
**Medium Emodin (40 mg/kg)**	73.7 ± 1.49	18.6 ± 1.77	1272.9 ± 65.02	98.8 ± 5.78	328 ± 57.04	569 ± 141.06	16.62 ± 0.17	7.78 ± 0.14	31.33 ± 0.37
**High Emodin (80 mg/kg)**	73.5 ± 1.28	18.3 ± 0.97	1268.7 ± 69.63	89.4 ± 4.04	332.3 ± 60.85	504.9 ± 102.89	16.67 ± 0.16	8.03 ± 0.20	32.1 ± 0.48

### Spleen and blood analyses do not indicate inflammatory responses to emodin feeding

**Fig. 3:**
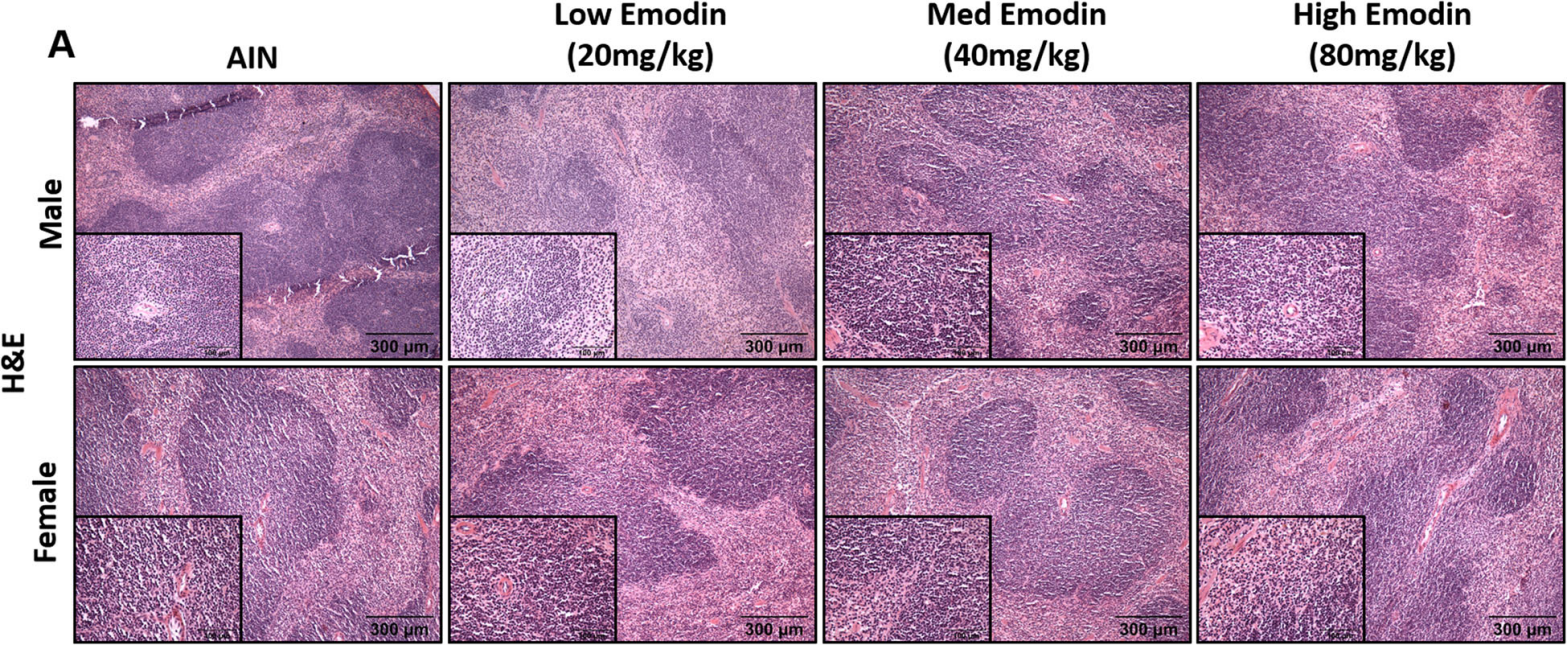
Twelve-week emodin feeding does not present histological changes in spleen. **a.** Representative 10X and 40X (insets) images of male and female spleens stained with H&E. *n* = 5/group

To assess the sub-chronic effect of emodin on circulating immune cells we performed a comprehensive blood panel analysis. Table 2 indicates that there was no effect of emodin at any dose on circulating immune cells. To further investigate the potential toxicity of emodin on the immune system, we performed H&E staining on spleen biopsies. There were no histological changes of red or white pulp and there appears to be no indication of blood flow blockage or fibrosis (Fig. 3a).

**Table 2:** Male and female mice fed emodin for 12-weeks do not show pertubations in CBC counts. White blood cells (WBC), lymphocytes (LYM), monocytes (MON), neutrophils (NEU), neutrophil:lymphoctye ratio (NLR), red blood cells (RBC), hemoglobin (HGB), hematocrit (HCT), and platelets (PLT) were measured using a VetScan HMT (Abaxis, Union City, CA). *n* = 5/group

Group	WBC (10^9/l)	LYM (10^9/l)	MON (10^9/l)	NEU (10^9/l)	NLR	RBC (10^12/l)	HGB (g/dl)	HCT (%)	PLT (10^9/l)
**Control (AIN)**	5.39 ± 0.63	4.03 ± 0.38	0.21 ± 0.06	1.15 ± 0.31	0.27 ± 0.06	10.41 ± 0.54	15.97 ± 0.15	42.06 ± 1.86	717.78 ± 62.73
**Low Emodin (20 mg/kg)**	4.99 ± 0.32	3.97 ± 0.26	0.17 ± 0.04	0.85 ± 0.10	0.21 ± 0.02	10.82 ± 0.10	16.39 ± 0.15	42.88 ± 0.43	716.90 ± 22.33
**Medium Emodin (40 mg/kg)**	5.30 ± 0.43	4.31 ± 0.29	0.14 ± 0.04	0.85 ± 0.16	0.19 ± 0.02	10.49 ± 0.14	16.39 ± 0.25	41.86 ± 0.59	680.20 ± 32.56
**High Emodin (80 mg/kg)**	5.58 ± 0.38	4.48 ± 0.31	0.18 ± 0.04	0.91 ± 0.12	0.21 ± 0.03	10.23 ± 0.17	16.03 ± 0.25	40.32 ± 0.67	693.60 ± 26.30

### Liver, kidney, and heart do not present with any noticeable toxicities after 12-wk emodin feeding

#### Emodin feeding does not cause liver toxicity

**Fig. 4:**
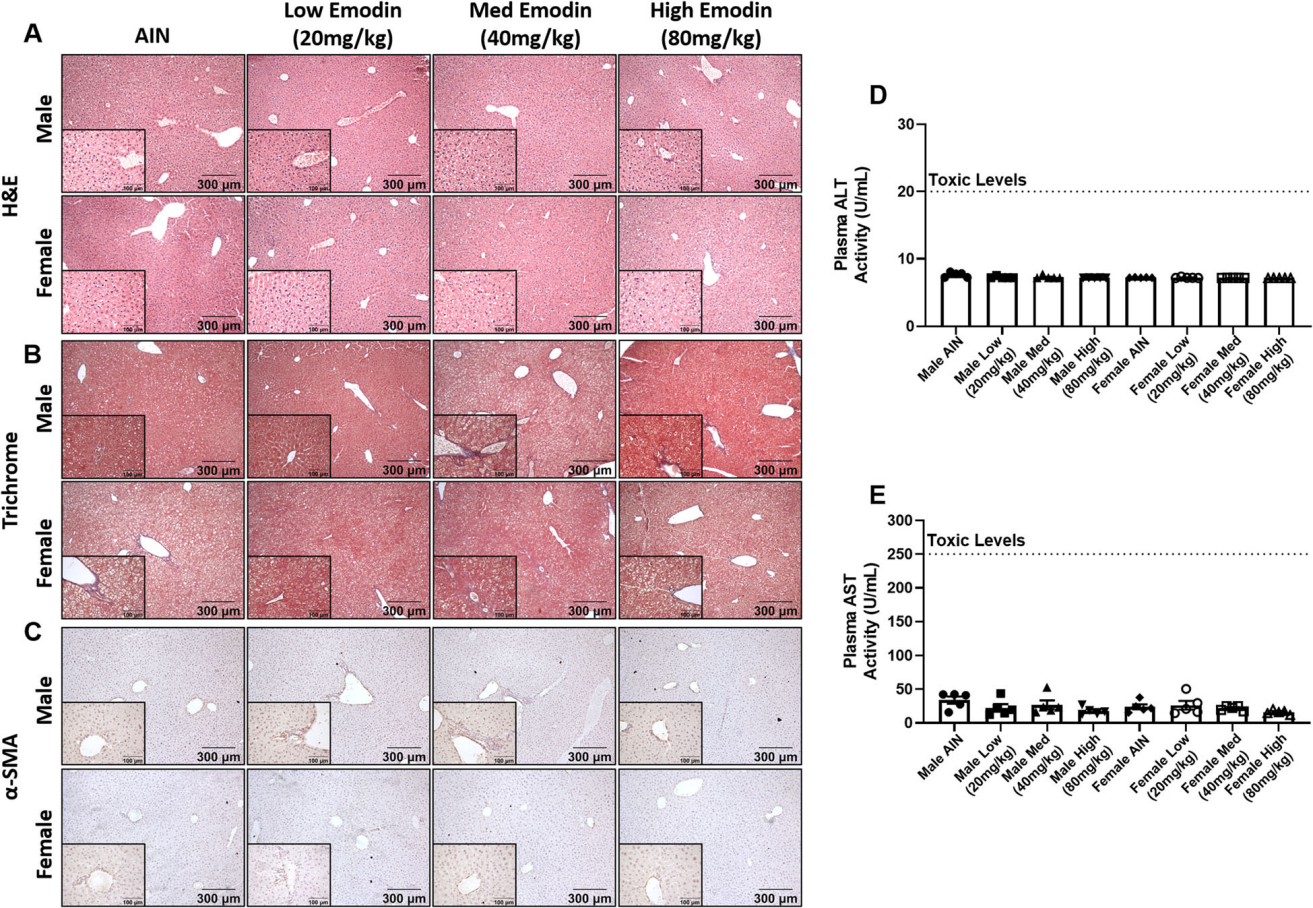
Twelve-week emodin feeding does not cause liver toxicity. **a.** Representative H&E stains of livers imaged at 10X and 40X (insets). **b.** Representative trichrome stains of livers imaged at 10X and 40X (insets). **c.** Representative images of livers stained with α-SMA imaged at 10X and 40X (insets) **d.** Plasma ALT activity measured in units/mL (U/mL) plasma. Dashed line indicates critical toxic levels (20 U/mL). **e**. Plasma AST activity measured in units/mL (U/mL) plasma. Dashed line indicates critical toxic levels (250 U/mL). *n* = 5/group

To investigate the potential toxicity of emodin to the liver we performed H&E and trichrome staining. There were no indications of fibrosis or NASH in any of the mice fed emodin diets (Fig. 4a-b). α-SMA staining was used to confirm these findings in liver specimens (Fig. 4c). Further, we measured ALT and AST levels in plasma. We found that there was no elevated plasma ALT or AST levels following any dose of emodin (Fig. 4d-e).

#### Emodin feeding does not cause kidney toxicity

**Fig. 5:**
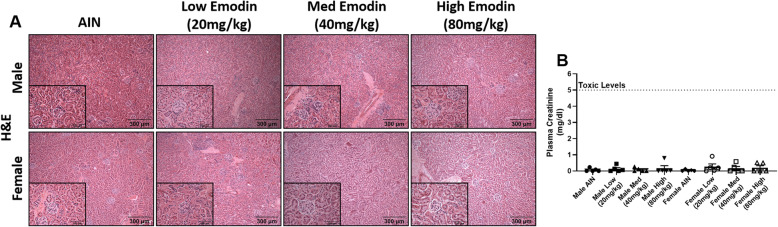
Twelve-week emodin feeding does not cause kidney toxicity. **a.** Representative H&E stains of kidneys imaged at 10X and 40X (insets). **b.** Plasma creatinine measured in milligrams/deciliters (mg/dl) of plasma. Dashed line indeicates critical toxic levels (5 mg/dl). *n* = 5/group

To investigate the potential toxicity of emodin to the kidneys we performed H&E and trichrome staining. There were very few indications (< 5%) of isolated tubular ectasia and sloughed cells in tubular lumens in addition to an absence of inflammation (Fig. 5a). To test the function of the kidneys we measured creatinine levels in plasma. Plasma creatinine levels were in normal range; lower that 5 mg/dl in any of the mice indicating normal renal function (Fig. 5c).

#### Emodin feeding does not cause noticeable toxicity to the cardiovascular system

**Fig. 6:**
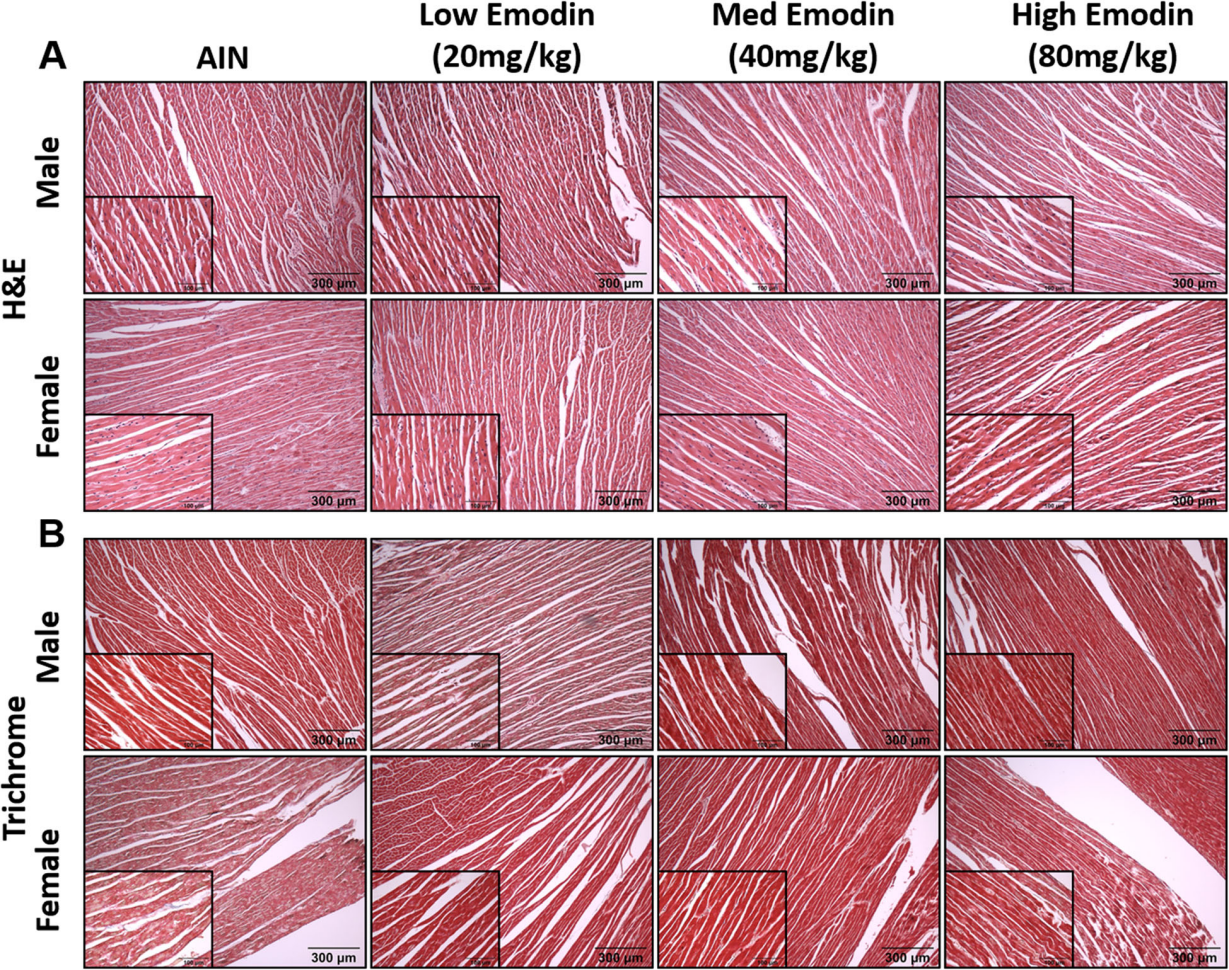
Twelve-week emodin feeding does not cause cardiotoxicity. **a.** Representative H&E stains of cardiac tissue imaged at 10X and 40X (insets). **b.** Representative trichrome stains of cardiac tissue imaged at 10X and 40X (insets)

To investigate the potential toxicity of emodin to the cardiovascular system, we performed H&E and trichrome staining on heart muscle (Fig. 6a-b). There was no indication of architectural change of the tissue or fibrosis in cardiac tissue. Further, we did not observe any indications of atrial or ventricular hypertrophy.

### Small intestine and colon analysis do not indicate GI toxicity after 12-weeks of emodin feeding

**Fig. 7:**
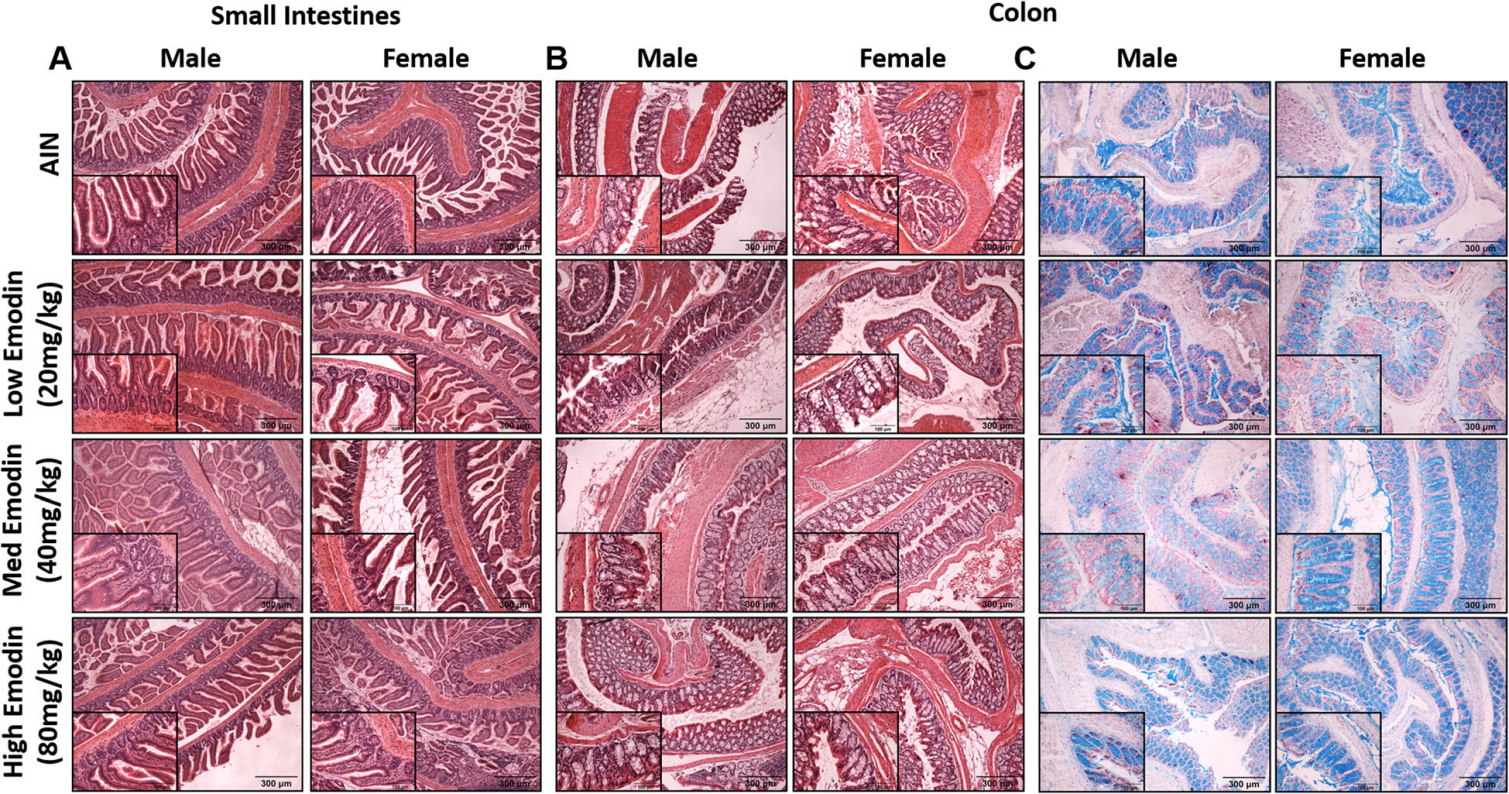
Twelve-week emodin feeding does not cause GI toxicity. **a.** Representative H&E stains of swiss rolled small intestines imaged at 10X and 40X (insets). **b.** Representative H&E stains of swiss rolled colons imaged at 10X and 40X (insets). **c.** Representative alcian blue stains of swiss rolled colons imaged at 10X and 40X (insets). *n* = 5/group

To assess potential gastrointestinal toxicities of sub-chronic emodin feeding, we performed histopathological analysis on small intestine (Fig. 7a) and colon tissue (Fig. 7b-c). H&E staining did not indicate any significant dysplasia in either the small intestine (Fig. 7a) or colon (Fig. 7b). We furthered stained colon biopsies with Alcian blue to assess goblet cell integrity. Alcian blue staining did not show any significant differences in goblet cell count or morphology in the colon (Fig. 7c).

## Discussion

Emodin, a natural anthraquinone, has shown many potential health benefits in pre-clinical models (Supplementary Table 1). In fact, we have reported that emodin is effective at reducing tumorigenesis given its actions on macrophages [14, 16]. However, further development of emodin as an effective anti-cancer agent is hindered by uncertainties surrounding its potential toxicity. The primary purpose of this study was to uncover any potential toxic properties of emodin in mice using doses that we have found to have efficacy in our cancer studies [14, 16]. In addition, we sought to assess the time course of emodin clearance when administered both I.P. and P.O. in order to begin to establish effective dosing intervals. We found that 12 weeks of low (20 mg/kg), medium (40 mg/kg), or high (80 mg/kg) emodin feeding does not cause pathophysiological perturbations in major organ systems. We also found that glucuronidated emodin peaks at 1 h for both I.P. and P.O. administered emodin and is eliminated by 12 h.

Despite inconsistencies in the literature, previous investigations have shown that emodin is typically cleared by 12 h after injection/ingestion [12, 16, 31]. We show in the present study that emodin is rapidly metabolized to its glucuronidated form, peaking at 1 h, and is completely cleared by 12 h when given both I.P. and P.O. Further, we confirm that the parent free emodin is undetectable after 1 h, even at higher doses. Some of the inconsistences in the literature are likely due to the varying vehicles used. It is important to note that emodin is not soluble solely in PBS or water and must have an alcohol-based solvent to dissolve. Several previous studies have reported using saline or PBS only as a vehicle [2, 5, 13, 32], which is likely to result in delivery of a non-homogenous solution, and presumably inconsistent pharmacokinetic data. We aimed to deliver a homogenous solution of emodin to improve our consistency of plasma analysis. Thus, emodin was dissolved in pure DMSO for the I.P. study and in propylene glycol for the P.O. study. Our findings are supported by previously published work [12, 16, 31, 33]. For example, a study by Shia et al., 2010 utilized a similar solvent to propylene glycol (PEG 400) and showed comparable results using male Sprague-Dawley rats [33]. While we report peak levels of emodin at 1 h post administration for both the I.P. and P.O. studies, it is important to note that our assessment was limited to 1 h, 4 h and 12 h time-points. Thus, it is certainly possible that emodin may have peaked prior to, or following, the 1 h time-point. Evaluation of additional time-points is necessary to fully determine the timing of peak levels of emodin.

Of further interest with the pharmacokinetic findings is the indication that females have almost double the amount of glucuronidated emodin in the circulation at 1 h following injection/gavage. When given I.P., this difference remains at the 4 h time-point (only significant in the 20 mg/kg dose), but not in mice given emodin via P.O. This finding is consistent with Liu et al., (2010) that showed in an ex vivo experiment that emodin is absorbed and metabolized at a faster rate in females vs. males [9]. Although the clearance of emodin is comparable between males and females, it is important to note this difference in pharmacokinetics between males and females so that appropriate dosing can be implemented during the transition to clinical investigations.

Given the potential toxicity associated with emodin along with our efforts in the development of emodin as a complementary cancer therapy we performed a sub-chronic toxicity study of emodin as a first-step to clinical translation. We subjected mice to an emodin infused diet of three different concentrations (~ 20 mg/kg, 40 mg/kg and 80 mg/kg) – similar doses that we have used in our efficacy studies - for 12 weeks to observe any potential toxic effects that emodin may have. Emodin did not impact body weight or body composition in the current study. Further, there was no effect of emodin on major organs. It should be noted that others have reported beneficial effects of emodin on metabolic disorders including a reduction in body weight; however, unlike the current study, these effects were documented in the settings of diet-induced obesity [34, 35].

There is some suggestion that emodin may have mutagenic properties given the documented reports of genotoxicity and mutagenicity in certain strains of bacteria [19, 20]. However, a two-year genetic toxicology and carcinogenesis study conducted by the National Toxicology Program (NTP) of the NCI showed no evidence of carcinogenic activity for emodin in female mice and equivocal evidence in male mice given a low incidence of renal tubule adenoma and carcinoma (1 mouse in each of 35 mg/kg and 70 mg/kg groups of 10) following a 2 year feeding study [36]. Only in doses of 170 mg/kg and higher were significant toxicities noticed in this study [36]. Together with other assessments of emodin from carcinogenicity studies, evidence does not support a genotoxic risk of emodin to humans [19, 20, 26]. In fact, there are a number of recent reports that indicate anti-tumor effects of emodin including studies by our group [14, 16]. This is further supported by the current study; we show that emodin, when given at doses of 20, 40, and 80 mg/kg for 12 weeks, does not appear to have mutagenic properties.

There also are some reports of hepatotoxic effects of emodin while other studies report hepatoprotection [21–25]. Based on a review of the literature it is likely that emodin has bidirectional potential, both liver protection [21, 22] and hepatoxicity [21–25] depending on the dose. In our study, we found no hepatoxicity with daily emodin treatment for 12 weeks and this was supported by lack of elevation of plasma ALT and AST levels. Further, liver specimens stained with α-SMA did not show any indications of liver fibrosis. Similarly, there are some indications that emodin, or foods containing emodin, may cause kidney toxicity [37] while other reports document that emodin may actually serve as a therapeutic for kidney injury [38]. We found no evidence of nephrotoxicity in the current study nor did we document elevated plasma creatinine levels following emodin treatment. Further, emodin has been shown to have laxative effects leading to melanosis, but only at very high doses, for example, 1–3 g/kg/d for mice [20, 26]. Thus, it is not surprising that we did not find any laxative effects following administration of emodin in the 20-80 mg/kg dose range. Nor did we document any dysplasia in the small intestine or the colon. While toxic effects of emodin have not been documented in the spleen or heart, at least to our knowledge, we assessed these tissues and report no toxic effects.

Our findings contribute to the literature on the safety of emodin in mice. Major strengths of our study include the use of male and female mice, administration of multiple doses of emodin, and assessment of both toxicity and pharmacokinetics in a single study. However, it also should be noted that there are several limitations to our study. We performed a sub-chronic toxicity study of emodin where assessments were performed at only one time point (i.e. 12 weeks of emodin). Chronic toxicity studies (i.e. 12 months or longer) with assessments performed at multiple time points would further add to the literature. In addition, we did not assess the LD50 of emodin and to our knowledge this has not been previously reported. However, in a comprehensive genotoxicity study performed by the National Toxicology Program doses of 800 mg/kg in male mice and 1100 mg/kg in female mice for 14 weeks did not lead to any mortality [36]. Similarly, in rats, 300 mg/kg daily for 14 weeks did not cause mortality. In their 2 year assessment (105 weeks) in mice and rats there was no increase in mortality following emodin administration [36]. Given published studies of emodin toxicity, it is likely that very large doses (excess of 1 g/kg) would need to be administered to mice in order to reach LD50. While we did not document any toxicity of emodin based on the histopathology assessment, additional outcomes should be evaluated in future studies to confirm these findings. For example, assessment of transforming growth factor β (TGF-β) to confirm liver fibrosis results. Interestingly, a recently published paper from our group has documented that emodin in fact decreases TGF-β production and signaling in macrophages and cancer cells [39]. Finally, we were not able to determine any mechanisms for the documented increase in glucuronidated emodin in female mice in the pharmacokinetic study. However, based on pervious work it has been established that emodin is absorbed and metabolized at a faster rate in females versus males [9]. Nonetheless, studies should further evaluate sex differences in emodin pharmacokinetics.

It is important to note that clinical data supporting the safety and pharmacokinetics of emodin in humans is lacking. Further, emodin does not currently have FDA approval. Thus, there may be unidentified disadvantages to the clinical use of emodin at this time. Currently, its use in humans appears to be largely limited to its laxative properties. As such, emodin supplements are currently available and marketed as a laxative. In addition, Morphogene Nutrition has marketed dietary supplement emodin as an advanced cortisol inhibitor with benefits in stress reduction, weight loss and microbial infection. However, clinical studies providing dosing for emodin for use as a treatment for constipation or other aliments are simply not available. It is certainly possible that higher doses of emodin in humans could lead to the same side effects that have been reported in experimental studies using large doses of emodin including mutagenic or hepatotoxic effects; although there is no evidence to support this at this time. Despite this, current consumers of emodin should be aware of the toxicity associated with large doses of emodin in the rodent literature. Thus, while our studies in rodents using doses of 20, 40, and 80 mg/kg appear to be safe, we cannot yet make conclusions on the safety of emodin in humans. Nonetheless, based on efficacy studies in rodents, emodin holds great promise as an anti-cancer agent. The current studies advance the clinical development of emodin as we report no toxicity of emodin at doses that we have shown to have anti-cancer efficacy. Further, while low bioavailability of emodin is evident, we have reported bioactivity in our cancer studies at these doses. In summary, the current study is a significant step in the clinical development of emodin as an anti-cancer agent.

## Conclusion

In summary, we show that the pharmacokinetics of emodin does not differ significantly between the peritoneal and oral routes of administration. However, there are sex differences in the pharmacokinetics of emodin with female mice having higher levels of metabolized emodin, likely due to the fact that emodin is absorbed and metabolized at a faster rate in females versus males [9], that should be considered during the transition to clinical investigations. Further, we confirm that emodin is a safe compound to be used in both male and female mice when given at 20, 40, and 80 mg/kg doses for 12 wks.

## Supplementary Information

**Additional file 1: Table S1.** Emodin Review Table. Emodin Review. Assessment of studies examining the potential therapeutic properties of emodin in various models of pathology. Studies that examined more than one dose of emodin or time point were treated as separate experiments when totaling each respective outcome.

## Data Availability

The datasets generated during and/or analyzed during the current study are available from the corresponding author on reasonable request.

## Abbreviations

ALT: Alanine Transaminase
α-SMA: Alpha-Smooth Muscle Actin
ANOVA: Analysis of Variance
AST: Aspartate Transaminase
BMC: Bone Mineral Content
BMD: Bone Mineral Density
DLAR: Department of Laboratory Animal Resources
DMSO: Dimethyl Sulfoxide
DEXA: Dual-Energy X-Ray Absorptiometry
EDTA: Ethylenediaminetetraacetic acid
Fig: Figure
g/kg/d: Grams Per Kilogram Per Day
H&E: Hematoxylin and Eosin
HCT: Hematocrit
Hb: Hemoglobin
I.P.: Intraperitoneal
LC-UV-MS: Liquid Chromatography Ultraviolet Mass Spectrometry
LC-MS/MS: Liquid Chromatography with Tandem Mass Spectrometry
LYM: Lymphocytes
mg: Milligrams
mm: Millimeters
mL: Milliliter
mg/kg: Milligrams Per Kilogram
MON: Monocytes
MRM: Multiple Reaction Monitoring
NEU: Neutrophils
NLR: Neutrophil/Lymphocyte Ratio
NASH: Nonalcoholic Steatohepatitis
NMR: Nuclear Magnetic Resonance
P.O: Oral Gavage
PBS: Phosphate Buffered Saline
PLT: Platelets
RBC: Red Blood Cells
SI: Small Intestine
SEM: Standard Error of Mean
TGF-β: Transforming Growth Factor β
U/mL: Units/mL
WBC: White Blood Cells
WT: Wildtype
