# Supplementary material for: Safety of natural anthraquinone emodin: an assessment in mice
Source: BMC Pharmacol Toxicol. 2021 Jan 28;22:9. doi: 10.1186/s40360-021-00474-1 (PMC7845031; doi:10.1186/s40360-021-00474-1)
Supplement: Supplementary file 1 — Additional file 1: Table S1. Emodin Review Table. Emodin Review. Assessment of studies examining the potential therapeutic properties of emodin in various models of pathology. Studies that examined more than one dose of emodin or time point were treated as separate experiments when totaling each respective outcome. [file 40360_2021_474_MOESM1_ESM.docx]

| *Supplementary Table 1. Emodin Review* | | | | | | | | | | | |
| --- | --- | --- | --- | --- | --- | --- | --- | --- | --- | --- | --- |
| Authors | Pathology Investigated | Experimental Model | Method of Pathology | Dose of Emodin | Route of Administration | Outcomes | | | | | |
|  |  |  |  |  |  | Overall Outcome | Body Weight | Inflammation/Pathology | Signaling | PK/PD | Gut Microbiota |
| Bai *et al*. (1) | Liver Cancer | BALB/c nu/nu mice (Male) | HepG2 Xenograft | 1 mg/kg or 10 mg/kg | Hypodermic Injection | Inhibition of tumorigenesis and reduced mortality | N/A | 10 mg/kg dose showed no mortality and reduced metastasis | SMAD2: ↓  SMAD4: ↓  miR-34a: ↑  VEGFR: ↓  AKT: ↓  ERK1/2: ↓ | Peak: 1hr  Cleared: 12 hr  t_1/2_: 2hr | N/A |
| Feng *et al*. (2) | Obesity and Metabolic Syndrome | C57BL/6J mice (Male) | HFD (60 kcal% fat, 20 kcal% protein, 20 kcal% carbohydrates) | 50 mg/kg or 100 mg/kg or 200 mg/kg | Daily Oral Gavage (Distilled H_2_O) | Improves body weight and reduces metabolic dysfunction | ↓ | Reduced blood glucose with Prednisone  Improved insulin sensitivity  Reduced cholesterol and triglycerides | 11βHSD1: ↓  PEPCK: ↓  G6Pase: ↓ | N/A | N/A |
| Guo *et al*.  (3) | Pancreatic Cancer | BALB/c nu/nu mice (Male) | PANC-1 Xenograft | 40 mg/kg and  80 mg/kg | I.P.  (0.9% Sodium Chloride) | Improves Gemcitabine Efficacy | ↑ compared to Gemcitabine Alone | Reduced NF-κB but no specific inflammatory markers were assayed | p-NF-κB: ↓  Survivin: ↓  XIAP: ↓  Caspase-9: ↑  Caspase-3: ↑ | N/A | N/A |
| Höhn *et al*. (4) | Colon Cancer | WAG/Rjj Rats (Male) | CC-531 | 2.5 mg/kg or 5 mg/kg | I.V. or I.P. (Saline) Daily | Anti-tumor effects when given in low doses | ↓ | N/A | N/A | Both routes had similar efficacies | N/A |
| Iwanowycz *et al*. (5) | Inflammation | *In-vitro* peritoneal MΦs | IL4, LPS, and IFNγ | 10 mg/ml | *In vitro* (DMSO) | Modulation of MΦ activity | N/A | Suppress M1 and M2 activity | Inhibited H3K27 trimethylation removal | N/A | N/A |
| Iwanowycz *et al*. (6) | Breast Cancer | C57BL/6 and BALB/c mice (Female) | EO771 and 4T1 Xenografts | 40 mg/kg | I.P. (2% DMSO) daily | Attenuated tumor growth | N/A | Inhibited MΦ infiltration  Increased T-cell activation | IRF4: ↓  p-STAT6: ↓  p-C/EBPβ: ↓  MCP1: ↓  CSF1: ↓  Thy-1: ↓ | N/A | N/A |
| Jelassi *et al.* (7) | Breast and non-small cell lung carcinomas | *In vitro* carcinoma | Breast  MCA-10A  MDA-MB-435  MDA-MB-468  Lung  A549 | 0.001 μM – 10 μM | *In vitro* (DMSO) | Inhibits cell invasion by antagonizing P2X7 receptors | N/A | N/A | Reduced P2X7 Receptor-mediated ATP current | N/A | N/A |
| Jia *et al.*  (8) | Obesity and Metabolic Syndrome | LDLR^-/-^ Mice (Female) | Western-type Diet | 40 mg/kg | I.P. with LPS once daily (up to 70 days) | Reduced NAFLD pathology | ↔ | Reduction of systemic and liver immune cell infiltration | p-Erk1/2: ↓  p-P38: ↓ | Peak: 1hr  Cleared: 24hr | N/A |
| Jia *et al*. (9) | Breast Cancer Metastasis | BALB/c and C57BL/6 mice (Female) | 4T1 and E0771 Xenografts | 40 mg/kg | I.P. (2% DMSO) daily | Reduced breast tumor metastasis and macrophage polarization | N/A | Reduced metastasis to lungs  Reduced MΦ migration  Suppressed M2 MΦ polarization | p-STAT6: ↓  p-C/EBPβ: ↓ | Peak: 1hr  Cleared: 24hr | N/A |
| Kuo *et al.* (10) | Glioma | *In vitro* carcinoma | C6 Glioma | 10-50 μM | *In vitro* (DMSO) | Attenuated cellular growth but induced drug resistance | N/A | N/A | ROS: ↓  Cellular Ca^2+^: ↓  p-NF-κB (24hr): ↓  p-NF-κB (48hr): ↑  Mdr1a: ↑ | N/A | N/A |
| Lee *et al*. (11) | Bone remodeling | *In vitro* osteoclasts | MC3T3-E1 mouse osteoblasts | 1 μM – 40μM | *In vitro* (DMSO) | Improve bone health and prevent osteoporosis | N/A | N/A | BMP-2: ↑  AP-1: ↑  p-PI3K: ↑  p-Akt: ↑  p-NF-κB: ↑  p-JNK: ↑  p-p38: ↑ | Toxic at 20 μM and above | N/A |
| Lee *et al*. (12) | Colorectal Cancer Tumorigenesis | *In vitro* carcinoma | HCT116, SW480, SNU-C2A, and SNU-C5 | 10 μM - 50 μM | *In vitro* (DMSO) | Reduced cell proliferation and increased protein ubiquitination | N/A | Reduced tumor cell growth and fatty acid synthase activity | FASN: ↓  LC3-II: ↓  Ubiquitin: ↑  p-Akt: ↓  p-PI3K: ↓  p-ERK1/2: ↑ | N/A | N/A |
| Liu *et al*. (13) | Chronic Inflammation | *In vitro* peritoneal MΦ (Wistar Rats) and HEK293 | ATP | 0.1 μM – 10 μM | *In vitro* (DMSO) | Inhibition of ATP-induced MΦ death | N/A | Expression of P2X_7_ receptor is essential | N/A | N/A | N/A |
| Liu *et al*. (14) | Pancreatic Cancer | *In vitro* carcinoma and BALB/c nu/nu mice (Male) | SW1990 (*In vitro* and xenograft) | *In vitro:*10-40 μM  *In vivo:* 20 mg/kg and 40 mg/kg | *In vitro* (DMSO)  *In vivo*: Intragastric intubation (DMSO) | Attenuated cellular growth and increased apoptosis | N/A | Reduced metastasis by 50% | p-NF-κB: ↓  Survivin: ↓  MMP-9: ↓  Cleaved Caspase-3: ↑ | N/A | N/A |
| Lu *et al*.  (15) | Anaphylaxis | *In vitro*: Bone marrow-derived mast cells (Balb/cJ) and HMC-1 cells  *In vivo*: ICR mice | *In vitro* and *In vivo*: DNP IgE and human serum albumin | *In vitro:*10-20 μM  *In vivo:* 5-40 mg/kg orally | *In vitro* (DMSO)  *In vivo*: Orally (DMSO) | Attenuation from mast cell-dependent anaphylactic reaction | N/A | Reduced mast cell degranulation | COX-2: ↓  IL-6: ↓  TNF-α: ↓  p-Akt: ↓  p-Iκκα/β: ↓  p-p65: ↓  Syk: ↓  LAT: ↓  PLCγ1: ↓ | N/A | N/A |
| Ma *et al.* (16) | Nasopharyngeal Carcinoma | *In vitro* carcinoma | CNE-2Z | 6.25 μM – 100 μM | *In vitro* (DMSO) | Attenuated cellular growth | N/A | N/A | Chloride channel blocker and tamoxifen block anti-tumor effect | N/A | N/A |
| Meng *et al*. (17) | Atherosclerosis | *In vitro* Sprague-Dawley primary RASMC | TNFα (10 ng/ml) | 0.1 μM – 10 μM | *In vitro* (DMSO) | Inhibited MMPs and Inflammatory Responses and improved cell viability | N/A | Improved cell migration and reduced inflammatory cytokines | MMP-2: ↓  MMP-9: ↓  IL1β: ↓  IL-6: ↓  ICAM-1: ↓  VCAM-1: ↓  MCP-1: ↓  CINC-2β: ↓  NF-κB: ↓ | N/A | N/A |
| Song *et al.* (18) | Autoimmune Myocarditis | Lewis Rats (Male) | Porcine cardiac myosin with Freund’s complete adjuvant and *Mycobacterium tuberculosis* H37Ra | 50 mg/kg | Intragastric intubation (DMSO) for 3 weeks | Improved ventricular function and reduced myocarditis | ↑ | Myocarditis: ↓  Heart Weight: ↓ | p-NF-κB: ↓  IL-1β: ↓  TNF-α: ↓ | N/A | N/A |
| Tang *et al*. (19) | Non-small cell lung carcinoma | *In vitro* carcinoma  *In vivo*: BALB/c nu/nu mice (female) | A549 and H1975 | *In vitro:*25, 50, and 75 μM  *In vivo:* 25 mg/kg and 50 mg/kg | *In vitro* (DMSO)  *In vivo* (DMSO) every other day | Reduced proliferation and invasion *in vitro* and at high dose reduced tumor size *in vivo* | N/A | Invasion: ↓  Inhibition of cell cycle arrest | PPARγ: ↓  p-ERK1/2: ↑  p-AMPKα: ↑ | N/A | N/A |
| Tzeng *et al*. (20) | Obesity | Wistar Rats (Male) | HFD (45% kcal% fat) | 40 mg/kg and 80 mg/kg | Daily Oral gavage (Distilled H_2_O) for 8 weeks | Reduced body weight and lipid profile | ↓ | Reduced hepatic lipid content and lipogenesis | p-AMPK: ↑  ACO: ↑  p-SREBP-1: ↑  FAS: ↑  CPT-1: ↑ | N/A | N/A |
| Wang *et al*. (21) | Gamma Radiation Toxicity | C57BL/6 Mice (Male) | 7Gy Radiation | 30 mg/kg daily | Not specified | Improved survival of mice undergoing gamma radiation | N/A | Increased villus height, crypt number, and goblet cell count in proximal jejunum | p53: ↓ | N/A | N/A |
| Wang *et al.* (22) | Allergic Rhinitis | C57BL/6 Mice (Female) | Ovalbumin-induced sensitivity | 10 mg/kg and 20 mg/kg | DMSO diluted to 0.1% | Reduced inflammatory reaction and inflammatory mediators | N/A | Reduced cellular infiltration to lungs and reduced IgE levels | IL-4: ↓  IL-5: ↓  IL-13: ↓  Eotaxin: ↓  MMP-9: ↓  HO-1: ↑ | N/A | N/A |
| Yang *et al*. (23) | Virus Transmission | Mosquito Species: *A. aegypti, A. togoi, and C. pipens pallens* | Blood from live mouse | 1 mg/L – 25 mg/L | Dissolved in blood | Caused death of 3 mosquito species | N/A | Will kill mosquitos before transmission of diseases | N/A | N/A | N/A |
| Yang *et al*. (24) | Liver Cancer | *In vitro* carcinoma | Bel-7402 HCC cell line | 100 μM | *In vitro* (DMSO) | Induced apoptosis in cell cultures | N/A | Reduced lipid metabolism in cell cultures | Caspase-3: ↑  Caspase-9: ↑  Cytochrome-C: ↑  AIF: ↑  Bax: ↑  Bcl-2: ↓ | N/A | N/A |
| Zeng *et al*. (25) | Chronic Kidney Disease | Sprague-Dawley rats (Male) | Chronic renal failure via nephrectomy | 1 mg/day | 5 ml colonic irrigation (0.4% CMC-Na) | Mitigation of renal injury and reduction of toxic bacteria | ↔ | Urea: ↓  Indoxyl sulfate: ↓  Creatinine: ↓ | N/A | N/A | Total Bacteria: ↔  Clostridium spp.: ↓  Lactobacillus spp.: ↑  Bifidbacteria spp.: ↑  Enterococcus spp.: ↓  C. perfringens: ↓  E. coli: ↓ |
| Zhang *et al*. (26) | Viral Myocarditis | *In vitro*: HL-1 and Human Cardiomyocytes  *In vivo*: A/J Mice (Male) | CVB3 Viral Infection | *In vitro*: 5 - 20 μM  *In vivo*: 40 mg/kg | *In vitro*: DMSO 30 min before infection  *In vivo*: I.P. (2% DMSO) 1hr before infection | Improved cell viability and survival | N/A | VP1: ↓ and reduced pfu count in heart | eEF2 (K366): ↓  eEF2 (T56): ↑  p-Akt: ↓  p70S6K: ↓  p-ERK1/2: ↓  p-p60RSK: ↑  p-mTORC1: ↓  p-4EBP1: ↓  L32: ↓ | N/A | N/A |
| Zhang *et al*. (27) | Acute Pancreatitis | HPDE6-C7 | ATP | 5.625 μM - 180 μM | *In vitro* (DMSO) | Reduced cell injury | N/A | Reduces the inflammatory response to ATP | P2X7: ↓  NLRP3: ↓  ASC: ↓  Caspase-1: ↓  IL-1β: ↓  IL-18: ↓ | N/A | N/A |

**Supplementary Table 1.** Emodin Review. Assessment of studies examining the potential therapeutic properties of emodin in various models of pathology. Studies that examined more than one dose of emodin or time point were treated as separate experiments when totaling each respective outcome. ↔ = No Change, ↓ = Decrease, ↑ = Increase, N/A = Not Assessed, HFD = High Fat Diet.

1. Bai J, Wu J, Tang R, Sun C, Ji J, Yin Z, et al. Emodin, a natural anthraquinone, suppresses liver cancer in vitro and in vivo by regulating VEGFR. Invest New Drugs. 2019 Apr. PubMed PMID: 30976957. Epub 2019/04/11. eng.

2. Feng Y, Huang SL, Dou W, Zhang S, Chen JH, Shen Y, et al. Emodin, a natural product, selectively inhibits 11beta-hydroxysteroid dehydrogenase type 1 and ameliorates metabolic disorder in diet-induced obese mice. Br J Pharmacol. 2010 Sep;161(1):113-26. PubMed PMID: 20718744. PMCID: PMC2962821. eng.

3. Guo HC, Bu HQ, Luo J, Wei WT, Liu DL, Chen H, et al. Emodin potentiates the antitumor effects of gemcitabine in PANC-1 pancreatic cancer xenograft model in vivo via inhibition of inhibitors of apoptosis. Int J Oncol. 2012 Jun;40(6):1849-57. PubMed PMID: 22378302. Epub 2012/02/29. eng.

4. Höhn P, Braumann C, Freiburger M, Koplin G, Dubiel W, Luu AM. Anti-tumorigenic Effects of Emodin and Its' Homologue BTB14431 on Vascularized Colonic Cancer in a Rat Model. Asian Pac J Cancer Prev. 2020 Jan;21(1):205-10. PubMed PMID: 31983185. Epub 2020/01/01. eng.

5. Iwanowycz S, Wang J, Altomare D, Hui Y, Fan D. Emodin Bidirectionally Modulates Macrophage Polarization and Epigenetically Regulates Macrophage Memory. J Biol Chem. 2016 May;291(22):11491-503. PubMed PMID: 27008857. PMCID: PMC4882421. Epub 2016/03/23. eng.

6. Iwanowycz S, Wang J, Hodge J, Wang Y, Yu F, Fan D. Emodin Inhibits Breast Cancer Growth by Blocking the Tumor-Promoting Feedforward Loop between Cancer Cells and Macrophages. Mol Cancer Ther. 2016 08;15(8):1931-42. PubMed PMID: 27196773. PMCID: PMC4975665. Epub 2016/05/18. eng.

7. Jelassi B, Anchelin M, Chamouton J, Cayuela ML, Clarysse L, Li J, et al. Anthraquinone emodin inhibits human cancer cell invasiveness by antagonizing P2X7 receptors. Carcinogenesis. 2013 Jul;34(7):1487-96. PubMed PMID: 23524196. Epub 2013/03/23. eng.

8. Jia X, Iwanowycz S, Wang J, Saaoud F, Yu F, Wang Y, et al. Emodin attenuates systemic and liver inflammation in hyperlipidemic mice administrated with lipopolysaccharides. Exp Biol Med (Maywood). 2014 Aug;239(8):1025-35. PubMed PMID: 24740873. PMCID: PMC4988953. Epub 2014/04/16. eng.

9. Jia X, Yu F, Wang J, Iwanowycz S, Saaoud F, Wang Y, et al. Emodin suppresses pulmonary metastasis of breast cancer accompanied with decreased macrophage recruitment and M2 polarization in the lungs. Breast Cancer Res Treat. 2014 Nov;148(2):291-302. PubMed PMID: 25311112. PMCID: PMC4224983. Epub 2014/10/14. eng.

10. Kuo TC, Yang JS, Lin MW, Hsu SC, Lin JJ, Lin HJ, et al. Emodin has cytotoxic and protective effects in rat C6 glioma cells: roles of Mdr1a and nuclear factor kappaB in cell survival. J Pharmacol Exp Ther. 2009 Sep;330(3):736-44. PubMed PMID: 19549930. Epub 2009/06/23. eng.

11. Lee SU, Shin HK, Min YK, Kim SH. Emodin accelerates osteoblast differentiation through phosphatidylinositol 3-kinase activation and bone morphogenetic protein-2 gene expression. Int Immunopharmacol. 2008 May;8(5):741-7. PubMed PMID: 18387517. Epub 2008/02/22. eng.

12. Lee KH, Lee MS, Cha EY, Sul JY, Lee JS, Kim JS, et al. Inhibitory effect of emodin on fatty acid synthase, colon cancer proliferation and apoptosis. Mol Med Rep. 2017 Apr;15(4):2163-73. PubMed PMID: 28260110. PMCID: PMC5364834. Epub 2017/02/28. eng.

13. Liu L, Zou J, Liu X, Jiang LH, Li J. Inhibition of ATP-induced macrophage death by emodin via antagonizing P2X7 receptor. Eur J Pharmacol. 2010 Aug;640(1-3):15-9. PubMed PMID: 20452342. Epub 2010/05/07. eng.

14. Liu A, Chen H, Wei W, Ye S, Liao W, Gong J, et al. Antiproliferative and antimetastatic effects of emodin on human pancreatic cancer. Oncol Rep. 2011 Jul;26(1):81-9. PubMed PMID: 21491088. Epub 2011/04/12. eng.

15. Lu Y, Yang JH, Li X, Hwangbo K, Hwang SL, Taketomi Y, et al. Emodin, a naturally occurring anthraquinone derivative, suppresses IgE-mediated anaphylactic reaction and mast cell activation. Biochem Pharmacol. 2011 Dec;82(11):1700-8. PubMed PMID: 21907188. Epub 2011/09/03. eng.

16. Ma L, Yang Y, Yin Z, Liu M, Wang L, Chen L, et al. Emodin suppresses the nasopharyngeal carcinoma cells by targeting the chloride channels. Biomed Pharmacother. 2017 Jun;90:615-25. PubMed PMID: 28411554. Epub 2017/04/12. eng.

17. Meng L, Yan D, Xu W, Ma J, Chen B, Feng H. Emodin inhibits tumor necrosis factor-α-induced migration and inflammatory responses in rat aortic smooth muscle cells. Int J Mol Med. 2012 Jun;29(6):999-1006. PubMed PMID: 22426603. Epub 2012/03/15. eng.

18. Song ZC, Wang ZS, Bai JH, Li Z, Hu J. Emodin, a naturally occurring anthraquinone, ameliorates experimental autoimmune myocarditis in rats. Tohoku J Exp Med. 2012 07;227(3):225-30. PubMed PMID: 22791134. eng.

19. Tang Q, Wu J, Zheng F, Hann SS, Chen Y. Emodin Increases Expression of Insulin-Like Growth Factor Binding Protein 1 through Activation of MEK/ERK/AMPKα and Interaction of PPARγ and Sp1 in Lung Cancer. Cell Physiol Biochem. 2017;41(1):339-57. PubMed PMID: 28214826. Epub 2017/01/26. eng.

20. Tzeng TF, Lu HJ, Liou SS, Chang CJ, Liu IM. Emodin, a Naturally Occurring Anthraquinone Derivative, Ameliorates Dyslipidemia by Activating AMP-Activated Protein Kinase in High-Fat-Diet-Fed Rats. Evid Based Complement Alternat Med. 2012;2012:781812. PubMed PMID: 22649478. PMCID: PMC3357974. Epub 2012/05/10. eng.

21. Wang J, Zhang Y, Zhu Q, Liu Y, Cheng H, Li T. Data on the radioprotective effect of emodin. Data Brief. 2017 Apr;11:290-5. PubMed PMID: 28275662. PMCID: PMC5328685. Epub 2016/12/30. eng.

22. Wang T, Zhong XG, Li YH, Jia X, Zhang SJ, Gao YS, et al. Protective effect of emodin against airway inflammation in the ovalbumin-induced mouse model. Chin J Integr Med. 2015 Jun;21(6):431-7. PubMed PMID: 25519442. Epub 2014/12/18. eng.

23. Yang YC, Lim MY, Lee HS. Emodin isolated from Cassia obtusifolia (Leguminosae) seed shows larvicidal activity against three mosquito species. J Agric Food Chem. 2003 Dec;51(26):7629-31. PubMed PMID: 14664519. eng.

24. Yang N, Li C, Li H, Liu M, Cai X, Cao F, et al. Emodin Induced SREBP1-Dependent and SREBP1-Independent Apoptosis in Hepatocellular Carcinoma Cells. Front Pharmacol. 2019;10:709. PubMed PMID: 31297058. PMCID: PMC6607744. Epub 2019/06/25. eng.

25. Zeng YQ, Dai Z, Lu F, Lu Z, Liu X, Chen C, et al. Emodin via colonic irrigation modulates gut microbiota and reduces uremic toxins in rats with chronic kidney disease. Oncotarget. 2016 Apr;7(14):17468-78. PubMed PMID: 27003359. PMCID: PMC4951226. eng.

26. Zhang HM, Wang F, Qiu Y, Ye X, Hanson P, Shen H, et al. Emodin inhibits coxsackievirus B3 replication via multiple signalling cascades leading to suppression of translation. Biochem J. 2016 Feb;473(4):473-85. PubMed PMID: 26621875. Epub 2015/11/30. eng.

27. Zhang Q, Hu F, Guo F, Zhou Q, Xiang H, Shang D. Emodin attenuates adenosine triphosphate‑induced pancreatic ductal cell injury in vitro via the inhibition of the P2X7/NLRP3 signaling pathway. Oncol Rep. 2019 Aug. PubMed PMID: 31524270. Epub 2019/08/08. eng.
